# Analysis of Flexural Vibrations of a Piezoelectric Semiconductor Nanoplate Driven by a Time-Harmonic Force

**DOI:** 10.3390/ma14143926

**Published:** 2021-07-14

**Authors:** Mengen Li, Qiaoyun Zhang, Bingbing Wang, Minghao Zhao

**Affiliations:** 1 School of Mechanics and Safety Engineering, Zhengzhou University, Zhengzhou 450001, China; lme70398581@163.com (M.L.); bbwang@zzu.edu.cn (B.W.); memhzhao@zzu.edu.cn (M.Z.); 2 Henan Key Engineering Laboratory for Anti-Fatigue Manufacturing Technology, Zhengzhou University, Zhengzhou 450001, China; 3 School of Mechanical and Power Engineering, Zhengzhou University, Zhengzhou 450001, China

**Keywords:** piezoelectric semiconductor, nanoplate, flexural vibration, natural frequency, vibration modal

## Abstract

The performance of devices fabricated from piezoelectric semiconductors, such as sensors and actuators in microelectromechanical systems, is superior; furthermore, plate structures are the core components of these smart devices. It is thus important to analyze the electromechanical coupling properties of piezoelectric semiconductor nanoplates. We established a nanoplate model for the piezoelectric semiconductor plate structure by extending the first-order shear deformation theory. The flexural vibrations of nanoplates subjected to a transversely time-harmonic force were investigated. The vibrational modes and natural frequencies were obtained by using the matrix eigenvalue solver in COMSOL Multiphysics 5.3a, and the convergence analysis was carried out to guarantee accurate results. In numerical cases, the tuning effect of the initial electron concentration on mechanics and electric properties is deeply discussed. The numerical results show that the initial electron concentration greatly affects the natural frequency and electromechanical fields of piezoelectric semiconductors, and a high initial electron concentration can reduce the electromechanical fields and the stiffness of piezoelectric semiconductors due to the electron screening effect. We analyzed the flexural vibration of typical piezoelectric semiconductor plate structures, which provide theoretical guidance for the development of new piezotronic devices.

## 1. Introduction

In 1960, Hutson discovered the piezoelectric effect in ZnO and CdS semiconductors [1]. However, owing to the weak piezoelectricity of piezoelectric semiconductors (PSCs), researchers early on usually treated them as normal semiconductor materials. With the development of micro and nanoscale fabrication technologies, PSC structures with larger piezoelectric effects were fabricated, such as ZnO fibers, films, bands, belts, spirals, and tubes [2,3,4]. Multifunctional devices now include sensing, driving, carrier transport, and photoelectron excitation in single PSC structures. Hence, PSCs enable considerable potential in new intelligent and multifunctional electronic devices [5,6,7,8]. However, basic problems in PSCs were found, such as electromechanical fields in fibers [9,10], near-field cracks [11,12], I–V characteristics of positive-negative carrier junctions [13,14], flexural and vibration of beams [15], thermal effects [16,17], and waves propagations [18,19].

In microelectromechanical systems, such as semiconductor devices, plate structures are the important core components. The theory studies on PSC plates also attracted much attention. For example, Yang and Zhou [20] derived two-dimensional (2D) equations coupled extensional, flexural, and thickness-shear motions of PSC thin plates from the three-dimensional equations by power series expansions in the plate thickness coordinate. They also analyzed the propagation of thickness-shear waves and the amplification effect of an electric field on thickness-shear waves. Similarly, Yang et al. [21] derived 2D equations coupled extensional, flexural, and thickness-shear motions of PSC laminated plates, and the amplification effect of an electric field on thickness-shear waves was analyzed. Li et al. [22] studied the thickness-extensional vibration of a piezoelectric semiconductor plate, the effect of semiconduction on mechanical-to-electrical energy conversion was investigated. Tian et al. [23] analyzed the characteristics of elastic waves in a PSC plate structure with Stroh theory, effects of the initial carrier density, plate thickness, and biasing electric field on the wave speed and attenuation were deeply discussed. Tian et al. [24] obtained analytical solutions of SH waves in transversely isotropic multilayered PSC plates and discussed the effect of the mechanical imperfect interface on the dispersion behavior of SH waves. Luo et al. [25] obtained the analytical solutions of electromechanical fields for an elastic and PSC laminated thin film with a pair of infinite opposite sides under a static flexural load and numerically investigated tuning effects of the initial electron concentration. Luo et al. [26] then studied the same PSC plate model as reference [25] under periodic loads and derived the first three-order natural frequencies. Zhao et al. [27] analyzed a thermal piezoelectric semiconductor plate with a shooting method and obtained the numerical solutions of the electromechanical field and temperature along a thickness-extensional direction.

During the service process, PSC devices are usually subjected to periodic loads. However, in current researches on PSC plates under periodic loads, PSC plates are restrictedly regarded as infinite plates. These steady vibration problems are then simplified as the one-dimensional (1D) extensional or flexural problems. Motivated by this, we derived the 2D equations of the finite PSC plate with coupled flexural and thickness-shear motions and investigated its flexural vibrations driven by a time-harmonic force. The modal analysis of the PSC plate was performed via COMSOL Multiphysics 5.3a. Natural frequencies and vibration modes of the electromechanical fields of the PSC plate were obtained, and the effect of the initial electron concentration on the vibrational properties was discussed. Basic equations for the PSCs plate are given in Section 2, and the forced vibration analysis is introduced in Section 3. The convergence analysis and numerical results are discussed in Section 4 and summarized in Section 5.

## 2. Piezoelectric Semiconductor Plate Model

For an *n*-type PSC without body force and free of electric charge, the three-dimensional (3D) basic theory can be described with Cartesian tensor notation. The equations of motion, Gauss’s law of electrostatics, and the equation of charge conservation can be written as [20]

(1) $$\begin{array}{l} \sigma_{ji,j}=\rho {\ddot{u}}_{i},\\ D_{i,i}=q\left(N_{D}^{+}-n\right),\;\;(i,j=1,2,3\;\mathrm{or}\;x,y,z),\\ J_{i,i}=q\dot{n}, \end{array}$$

where *σ_ji_*, *D_i_*, and *J_i_* are the stress tensor, electric displacement component, and electric current density component, respectively; *ρ* and *u_i_* denote the mass density and displacement components, respectively; *q*, $N_{D}^{+}$, and *n* denote the unit electric charge (1.602 × 10^−19^ C), donor impurity and electron concentrations, respectively. Moreover, the comma in the subscript indicates the partial differentiation, ${\ddot{u}}_{i}$ and $\dot{n}$ respectively denote the 2-order and 1-order partial differentiation with respect to the time independent *t*.

The constitutive relations for 3D PSCs are given by:

(2) $$\begin{array}{l} \sigma_{ij}=c_{ijkl}\varepsilon_{kl}-e_{kij}E_{k},\\ D_{i}=e_{ikl}\varepsilon_{kl}+\kappa_{ik}E_{k},\\ J_{i}=qn\mu_{ij}E_{j}+qD_{ij}n_{,j}, \end{array}$$

where *ε_kl_* and *E_k_* are the strain tensor and electric field components, respectively, *c_ijkl_*, *e_kij_*, and *κ_ik_* are the elastic, piezoelectric, and dielectric constants, respectively, and *μ_ij_* and *D_ij_* are the electron mobility and diffusion coefficients, respectively. The strains *ε_ij_* and electric fields *E_i_* are related to the displacement *u_i_* and the electric potential *Φ_i_* through

(3) $$\begin{array}{l} \varepsilon_{ij}=(u_{i.j}+u_{j,i})/2,\\ E_{i}=-\phi_{,i}. \end{array}$$

The electron concentration *n* can be written as *n* = *n*_0_ + Δ*n*, where Δ*n* is the electron concentration perturbation, *n*_0_ is the initial electron concentration. For a small electron concentration perturbation, the constitutive relations in Equation (2) can be linearized as

(4) $$\begin{array}{l} \sigma_{ij}=c_{ijkl}\varepsilon_{kl}-e_{kij}E_{k},\\ D_{i}=e_{ikl}\varepsilon_{kl}+\kappa_{ik}E_{k},\\ J_{i}=qn_{0}\mu_{ij}E_{j}+qD_{ij}{\left(\Delta n\right)}_{,j}, \end{array}$$

In a natural state, $n_{0}=N_{D}^{+}$, thus, Equation (1) becomes

(5) $$\begin{array}{l} \sigma_{ji,j}=\rho {\ddot{u}}_{i},\\ D_{i,i}=-q\Delta n,\\ J_{i,i}=q\Delta \dot{n}. \end{array}$$

**Figure 1:**
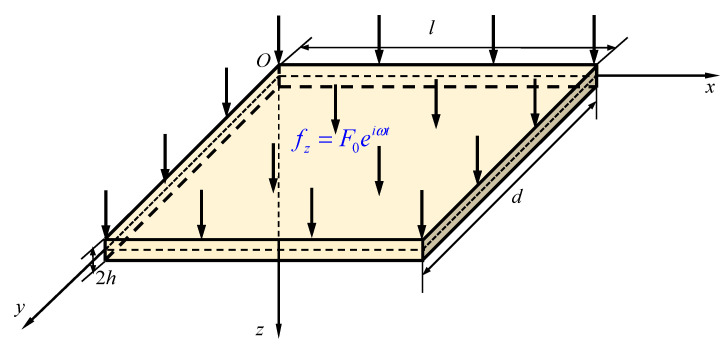
A PSC plate subjected to a transverse time-harmonic force *f_z_*.

We now consider a transversely isotropic PSC plate with thickness 2 *h*, length *l*, and width *d* (2 *h* < < *l*, *d*) under a transversely time-harmonic force *f_z_*, the reference coordinate plane *o*-*xy* is in the geometric middle plane of the plate, as depicted in Figure 1.

To overcome the complexity of a 3D PSC plate and describe the transient behaviors correctly, we simplify it to a 2D PSC plate model by extending the first-order shear deformation theory, in which shear and flexural motions in the plate thickness direction are considered. The mechanical displacements, electric potential, and electron concentration perturbation are approximated by [20]

(6) $$\begin{array}{l} u_{x}≅z\psi_{x}(x,y,t),\;\\ u_{y}≅z\psi_{y}(x,y,t),\\ u_{z}≅w(x,y,t),\\ \phi ≅\phi (x,y,t),\\ \Delta n≅\Delta n(x,y,t), \end{array}$$

where *u_x_*, *u_y_*, and *u_z_* are the mechanical displacements, *Ψ_x_* and *Ψ_y_* are the plate-thickness shear displacements, and *w* is the deflection. Substitution of Equation (6) into Equation (3), the relevant strains can be expressed as

(7) $$\begin{array}{l} \varepsilon_{x}=\frac{\partial u_{x}}{\partial x}≅z\frac{\partial \psi_{x}}{\partial x},\;\\ \varepsilon_{y}=\frac{\partial u_{y}}{\partial y}≅z\frac{\partial \psi_{y}}{\partial y},\;\\ \gamma_{xy}=\frac{\partial u_{x}}{\partial y}+\frac{\partial u_{y}}{\partial x}≅z(\frac{\partial \psi_{x}}{\partial y}+\frac{\partial \psi_{y}}{\partial x}),\\ \gamma_{zx}=\frac{\partial u_{x}}{\partial z}+\frac{\partial u_{z}}{\partial x}≅\psi_{x}+\frac{\partial w}{\partial x},\\ \gamma_{zy}=\frac{\partial u_{y}}{\partial z}+\frac{\partial u_{z}}{\partial y}≅\psi_{y}+\frac{\partial w}{\partial y}. \end{array}$$

By introducing the stress relaxation approximation of *σ_z_* = 0 into Equation (2), we have the following expression

(8) $$\varepsilon_{z}=-\left(c_{33kl}\varepsilon_{kl}-c_{3333}\varepsilon_{33}-e_{k33}E_{k}\right)/c_{3333}$$

By substituting Equations (7) and (8) into Equation (4), the constitutive equations can be rewritten as

(9) $$\begin{array}{l} \sigma_{x}=z({\bar{c}}_{11}\frac{\partial \psi_{x}}{\partial x}+{\bar{c}}_{12}\frac{\partial \psi_{y}}{\partial y}),\\ \sigma_{y}=z({\bar{c}}_{12}\frac{\partial \psi_{x}}{\partial x}+{\bar{c}}_{11}\frac{\partial \psi_{y}}{\partial y}),\\ \tau_{xy}=c_{66}z(\frac{\partial \psi_{x}}{\partial y}+\frac{\partial \psi_{y}}{\partial x}),\\ \tau_{zx}=c_{44}(\psi_{x}+\frac{\partial w}{\partial x})+e_{15}\frac{\partial \phi }{\partial x},\\ \tau_{zy}=c_{44}(\psi_{y}+\frac{\partial w}{\partial y})+e_{15}\frac{\partial \phi }{\partial y},\\ D_{x}=e_{15}(\psi_{x}+\frac{\partial w}{\partial x})-\kappa_{11}\frac{\partial \phi }{\partial x},\\ D_{y}=e_{15}(\psi_{y}+\frac{\partial w}{\partial y})-\kappa_{11}\frac{\partial \phi }{\partial y},\\ J_{x}=-qn_{0}\mu_{11}\frac{\partial \phi }{\partial x}+qD_{11}\frac{\partial \Delta n}{\partial x},\\ J_{y}=-qn_{0}\mu_{11}\frac{\partial \phi }{\partial y}+qD_{11}\frac{\partial \Delta n}{\partial y}, \end{array}$$

where the effective material constants are defined by

(10) $${\bar{c}}_{11}=\frac{c_{11}-c_{13}^{2}}{c_{33}},\;{\bar{c}}_{12}=\frac{c_{12}-c_{13}^{2}}{c_{33}}.$$

To make the 2D plate model yield the same natural frequencies as the 3D PSC structure, two shear correction factors *k*_1_ and *k*_2_ must be introduced to moderate the excessive transverse shear strain energy. The replaced strains *γ_xz_* and *γ_zy_* are written as [20]

(11) $$\gamma_{zx}\to k_{1}\gamma_{zx},\;\gamma_{zy}\to k_{2}\gamma_{zy}.$$

Integrating Equation (9) through the thickness, the extended inner forces of the PSC plate are defined by

(12) $$\begin{array}{l} M_{x}=\int_{-h}^{h}z\sigma_{x}dz,\;\;\;M_{y}=\int_{-h}^{h}z\sigma_{y}dz\;,\;\;\;M_{xy}=\int_{-h}^{h}z\tau_{xy}dz,\;\\ \;Q_{zx}=\int_{-h}^{h}\tau_{zx}dz,\;\;\;Q_{zy}=\int_{-h}^{h}\tau_{zy}dz,\\ d_{x}=\int_{-h}^{h}D_{x}dz,\;\;\;d_{y}=\int_{-h}^{h}D_{y}dz,\;\;\;\;\\ j_{x}=\int_{-h}^{h}J_{x}dz,\;\;\;\;j_{y}=\int_{-h}^{h}J_{y}dz,\;\;\; \end{array}$$

where *M_x_*, *M_y_*, and *M_xy_* are the bending moments and torque, *Q_zx_* and *Q_zy_* are shear stresses, *d_x_* and *d_y_* are surface electric charges, and *j_x_* and *j_y_* are surface electric current densities. Then, we have

(13) $$\begin{array}{l} M_{x}=\frac{2}{3}h^{3}({\bar{c}}_{11}\frac{\partial \psi_{x}}{\partial x}+{\bar{c}}_{12}\frac{\partial \psi_{y}}{\partial y}),\\ M_{y}=\frac{2}{3}h^{3}({\bar{c}}_{12}\frac{\partial \psi_{x}}{\partial x}+{\bar{c}}_{11}\frac{\partial \psi_{y}}{\partial y}),\\ M_{xy}=\frac{2}{3}h^{3}c_{66}(\frac{\partial \psi_{x}}{\partial y}+\frac{\partial \psi_{y}}{\partial x}),\\ Q_{zx}=2h\;[k_{1}^{2}c_{44}(\psi_{x}+\frac{\partial w}{\partial x})+k_{1}e_{15}\frac{\partial \phi }{\partial x}],\\ Q_{zy}=2h\;[k_{2}^{2}c_{44}(\psi_{y}+\frac{\partial w}{\partial y})+k_{2}e_{15}\frac{\partial \phi }{\partial y}],\\ d_{x}=2h\;[k_{1}e_{15}(\psi_{x}+\frac{\partial w}{\partial x})-\kappa_{11}\frac{\partial \phi }{\partial x}],\\ d_{y}=2h\;[k_{2}e_{15}(\psi_{y}+\frac{\partial w}{\partial y})-\kappa_{11}\frac{\partial \phi }{\partial y}],\\ j_{x}=2hq(-n_{0}\mu_{11}\frac{\partial \phi }{\partial x}+D_{11}\frac{\partial \Delta n}{\partial x}),\\ j_{y}=2hq(-n_{0}\mu_{11}\frac{\partial \phi }{\partial y}+D_{11}\frac{\partial \Delta n}{\partial y}). \end{array}$$

By integrating Equation (5) with *z* through the plate thickness, the equations of shear and flexural motions, Gauss’s law, and charge conservation for the 2D plate model are given by

(14) $$\begin{array}{l} \frac{\partial M_{x}}{\partial x}+\frac{\partial M_{xy}}{\partial y}-Q_{zx}+f_{x}=\frac{2\rho h^{3}}{3}\frac{\partial^{2}\psi_{x}}{\partial t^{2}},\\ \frac{\partial M_{xy}}{\partial x}+\frac{\partial M_{y}}{\partial y}-Q_{zy}+f_{y}=\frac{2\rho h^{3}}{3}\frac{\partial^{2}\psi_{y}}{\partial t^{2}},\\ \frac{\partial Q_{zx}}{\partial x}+\frac{\partial Q_{zy}}{\partial y}+f_{z}=2h\rho \frac{\partial^{2}w}{\partial t^{2}},\\ \frac{\partial d_{x}}{\partial x}+\frac{\partial d_{y}}{\partial y}+\varpi =-2hq\Delta n,\\ \frac{\partial j_{x}}{\partial x}+\frac{\partial j_{y}}{\partial y}+\vartheta =2hq\frac{\partial \Delta n}{\partial t}, \end{array}$$

where *f_x_*, *f_y_*, *f_z_*, *ϖ*, and *ϑ* are the equivalent surface loads, surface electric charge, and surface electric current density, respectively. They are defined by

(15) $$f_{x}={\left[z\tau_{zx}\right]}_{-h}^{h},\;f_{y}={\left[z\tau_{zy}\right]}_{-h}^{h},\;f_{z}={\left[\sigma_{z}\right]}_{-h}^{h},\;\;\varpi ={\left[D_{z}\right]}_{-h}^{h},\;\vartheta ={\left[J_{z}\right]}_{-h}^{h}.\;\;\;\;$$

When the plate is subjected to a transverse time-harmonic force *f_z_*, then *f_x_* = *f_y_* = 0, *ϖ* = 0, and *ϑ* = 0. The substitution of Equation (13) into Equation (14) yields the governing equations for the PSC plate

(16) $$\begin{array}{l} {\bar{c}}_{11}\frac{\partial^{2}\psi_{x}}{\partial x^{2}}+c_{66}\frac{\partial^{2}\psi_{x}}{\partial y^{2}}+\left({\bar{c}}_{12}+c_{66}\right)\frac{\partial^{2}\psi_{y}}{\partial x\partial y}-3h^{-2}k2c_{44}\left(\psi_{x}+\frac{\partial w}{\partial x}\right)-3h^{-2}ke_{15}\frac{\partial \phi }{\partial x}=\rho \frac{\partial^{2}\psi_{x}}{\partial t^{2}},\\ c_{66}\frac{\partial^{2}\psi_{y}}{\partial x^{2}}+{\bar{c}}_{11}\frac{\partial^{2}\psi_{y}}{\partial y^{2}}+\left({\bar{c}}_{12}+c_{66}\right)\frac{\partial^{2}\psi_{x}}{\partial x\partial y}-3h^{-2}k^{2}c_{44}\left(\psi_{y}+\frac{\partial w}{\partial y}\right)-3h^{-2}ke_{15}\frac{\partial \phi }{\partial y}=\rho \frac{\partial^{2}\psi_{y}}{\partial t^{2}},\\ k^{2}c_{44}\left(\frac{\partial \psi_{x}}{\partial x}+\frac{\partial^{2}w}{\partial x^{2}}\right)+ke_{15}\frac{\partial^{2}\phi }{\partial x^{2}}+k^{2}c_{44}\left(\frac{\partial \psi_{y}}{\partial y}+\frac{\partial^{2}w}{\partial y^{2}}\right)+ke_{15}\frac{\partial^{2}\phi }{\partial y^{2}}+f_{z}=\rho \frac{\partial^{2}w}{\partial t^{2}},\\ -\kappa_{11}\frac{\partial^{2}\phi }{\partial x^{2}}+ke_{15}\left(\frac{\partial \psi_{x}}{\partial x}+\frac{\partial^{2}w}{\partial x^{2}}\right)-\kappa_{11}\frac{\partial^{2}\phi }{\partial y^{2}}+ke_{15}\left(\frac{\partial \psi_{y}}{\partial y}+\frac{\partial^{2}w}{\partial y^{2}}\right)=-q\Delta n,\\ -n_{0}\mu_{11}\frac{\partial^{2}\phi }{\partial x^{2}}+D_{11}\frac{\partial^{2}\Delta n}{\partial x^{2}}-n_{0}\mu_{11}\frac{\partial^{2}\phi }{\partial y^{2}}+D_{11}\frac{\partial^{2}\Delta n}{\partial y^{2}}=\frac{\partial \Delta n}{\partial t}. \end{array}$$

It is assumed that the four plate edges are fixed, and that contact between metals and the PSC plate is ohmic. Then, the boundary conditions for the PSC plate are given by

(17) $$\begin{array}{l} \psi_{x}=0,\;\psi_{y}=0,\;w=0,\;\phi =0,\;\Delta n=0,\;\;x=0\;\;\mathrm{and}\;\;l,\\ \psi_{x}=0,\;\psi_{y}=0,\;w=0,\;\phi =0,\;\Delta n=0,\;\;y=0\;\;\mathrm{and}\;\;d. \end{array}$$

In addition, the initial state of the PSC plate is considered static, that is

(18) $$\begin{array}{l} \psi_{x}=0,\;\psi_{y}=0\;w=0,\;\Delta n=0,\\ \;\;\;\;\;\;\;\;\;\;\;\;\;\;\;\;\;\;\;\;\;\;\;\;\;\;\;\;\;\;\;\;\;\;\;\;\;\;\;\;\;\;\;\;\;\;\;\;\;\;\;\;\;\;\;\;t=0\\ \frac{\partial \psi_{x}}{\partial t}=0,\;\;\frac{\partial \psi_{y}}{\partial t}=0,\;\;\;\frac{\partial w}{\partial t}=0, \end{array}$$

## 3. Modal Analysis

As shown in Figure 1, the upper surface of the PSC plate is under a transverse time-harmonic force *f_z_* = *F*_0_ e*^i^^ωt^*, where *ω* is the excitation frequency. For harmonic motion, the solutions of the governing equations are

(19) $$\left\{\begin{array}{c} \psi_{x}\\ \psi_{y}\\ w\\ \phi \\ \Delta n \end{array}\right\}=\left\{\begin{array}{c} \Psi_{x}\\ \Psi_{y}\\ W\\ \Phi \\ \Delta N \end{array}\right\}e^{i\omega t},$$

where *Ψ_x_*, *Ψ_y_*, *W*, *Φ*, and Δ*N* are extended mode shapes. By substitution of Equation (19) into Equation (16), the common factor of *e^iωt^* can be canceled from the differential equations Equation (16), then governing equations for *Ψ_x_*, *Ψ_y_*, *W*, *Φ*, and Δ*N* can be rewritten as

(20) $$\begin{array}{l} \rho \omega^{2}\Psi_{x}+{\bar{c}}_{11}\frac{\partial^{2}\Psi_{x}}{\partial x^{2}}+c_{66}\frac{\partial^{2}\Psi_{x}}{\partial y^{2}}+({\bar{c}}_{12}+c_{66})\frac{\partial^{2}\Psi_{y}}{\partial x\partial y}-3h^{-2}k^{2}c_{44}(\Psi_{x}+\frac{\partial W}{\partial x})-3h^{-2}ke_{15}\frac{\partial \Phi }{\partial x}=0,\\ \rho \omega^{2}\Psi_{y}+c_{66}\frac{\partial^{2}\Psi_{y}}{\partial x^{2}}+{\bar{c}}_{11}\frac{\partial^{2}\Psi_{y}}{\partial y^{2}}+({\bar{c}}_{12}+c_{66})\frac{\partial^{2}\Psi_{x}}{\partial x\partial y}-3h^{-2}k^{2}c_{44}(\Psi_{y}+\frac{\partial W}{\partial y})-3h^{-2}ke_{15}\frac{\partial \Phi }{\partial y}=0,\\ \rho \omega^{2}W+k^{2}c_{44}(\frac{\partial \Psi_{x}}{\partial x}+\frac{\partial^{2}W}{\partial x^{2}})+ke_{15}\frac{\partial^{2}\Phi }{\partial x^{2}}+k^{2}c_{44}(\frac{\partial \Psi_{y}}{\partial y}+\frac{\partial^{2}W}{\partial y^{2}})+ke_{15}\frac{\partial^{2}\Phi }{\partial y^{2}}+F_{0}=0,\\ -\kappa_{11}\frac{\partial^{2}\Phi }{\partial x^{2}}+ke_{15}(\frac{\partial \Psi_{x}}{\partial x}+\frac{\partial^{2}W}{\partial x^{2}})-\kappa_{11}\frac{\partial^{2}\Phi }{\partial y^{2}}+ke_{15}(\frac{\partial \Psi_{y}}{\partial y}+\frac{\partial^{2}W}{\partial y^{2}})=-q\Delta N,\\ -i\omega \Delta N-n_{0}\mu_{11}\frac{\partial^{2}\Phi }{\partial x^{2}}+D_{11}\frac{\partial^{2}\Delta N}{\partial x^{2}}-n_{0}\mu_{11}\frac{\partial^{2}\Phi }{\partial y^{2}}+D_{11}\frac{\partial^{2}\Delta N}{\partial y^{2}}=0. \end{array}$$

The Formula (20) is a partial differential equation set that is hard to solve analytically with boundary conditions in Formula (17); therefore, the advanced numerical simulation software COMSOL Multiphysics (version 5.3a) is chosen to solve this steady vibration problem in PSC plate. With the use of the eigenvalue solver, vibration modals including nature frequencies *ω* and extended vibration modes (*Ψ_x_*, *Ψ_y_*, *W*, *Φ*, and Δ*N*) can be derived, and the extended internal forces (*M_x_*, *M_y_*, *M_xy_*, *Q_zx_*, *Q_zy_*, *d_x_*, *d_y_*, *j_x_*, and *j_y_*) can also be derived.

## 4. Numerical Examples

As one kind of third-generation semiconductor material, gallium nitride (GaN) is widely used in new types of intelligent and multifunctional electronic devices due to its wide bandgap, high piezoelectric, and other excellent functional properties. Therefore, in the following numerical cases, a GaN plate with a thickness of 2 *h* = 1 μm, length of *l* = 10 μm, and width of *d* = 10 μm is examined. The material constants of the GaN plate are listed in Table 1 [28], and the shear correction factors *k*_1_ and *k*_2_ are set as *k*_1_= *k*_2_ = 0.9069 [20]. We assume that the applied time-harmonic force *f_z_* is a sine wave, and its amplitude is a constant as *F*_0_ = 10^3^ N/m^2^.

### 4.1. Vibration Behaviors

The initial electron concentration *n*_0_ was fixed at 10^20^ m^−3^ to ensure converged numerical results. The extended vibration modes (*W*, *Φ*, and Δ*N*) of the central point (*l*/2, *d*/2) in the plate versus the total element number *N_E_* were calculated (see Table 2). In the following calculation, we used *N_E_* = 300 × 300 to discretize the PSC plate in consideration of both the calculation accuracy and efficiency.

**Figure 2:**
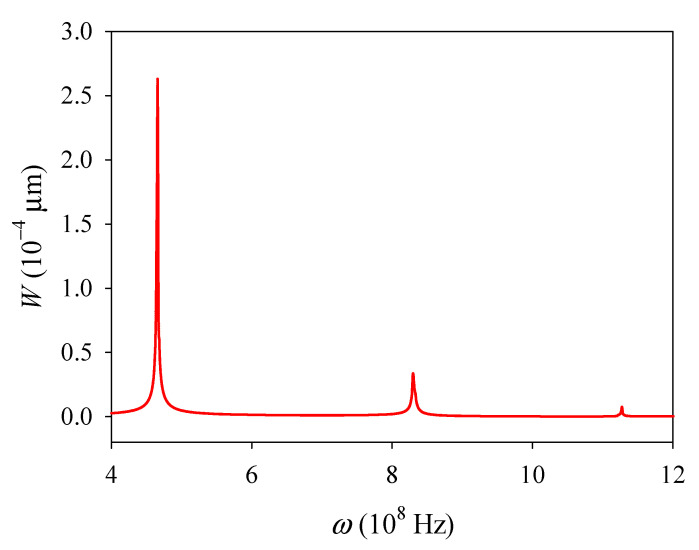
Deflection *W* versus the driving frequency *ω*.

For the central point (*l*/2, *d*/2) of the plate, the absolute value of the deflection *W* versus the excitation frequency ω is plotted in Figure 2. Three peaks *W* occur because of the resonance and correspond to the first three order natural frequencies *ω*_1_, *ω*_2_, and *ω*_3_, with values 4.653 × 10^8^ rad/s, 8.305 × 10^8^ rad/s, and 11.265 × 10^8^ rad/s, respectively. In addition, the electric potential and electron concentration perturbation corresponding to resonance frequencies also reaches their peaks. In general, a high energy conversion efficiency for conversing mechanical energy into electrical energy can be realized in a resonance state; this can be used in the piezoelectric vibration energy harvesters.

**Figure 3:**
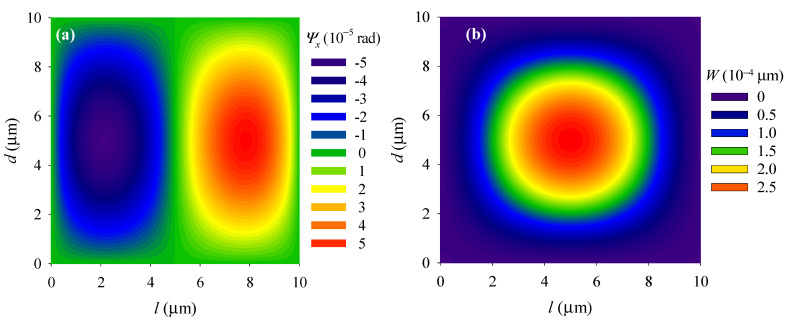

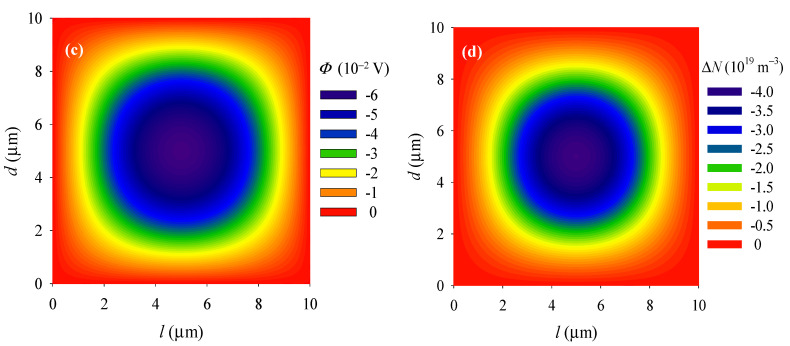
Distributions of (**a**) shear displacement Ψ*_x_*, (**b**) deflection *W*, (**c**) electric potential Φ, and (**d**) electron concentration perturbation Δ*N* in the plate.

We then used the first-order natural frequency as the driving frequency (*ω* = *ω*_1_) and examined the first-order modal of the PSC plate. Due to the symmetry, all the distribution patterns of the electromechanical fields along the *x*-direction are the same as those along the *y*-direction. Therefore, only the electromechanical fields along the *x*-direction were analyzed. Shear displacement *ψ_x_* is antisymmetrically distributed around the central line *x* = *l*/2 (see Figure 3a), and extreme values occur at points (*l*/4, *d*/2) and (3 *l*/4, *d*/2). The distributions of the deflection *W*, electric potential Φ, and electron concentration perturbation Δ*N* are similar, and all change uniformly. Extreme values all occur at the central point (see Figure 3b–d) due to the uniformly distributed load and the fixed boundary conditions. In addition, in Figure 3d, an electron redistribution phenomenon can be clearly observed. This phenomenon occurs due to electrons spontaneously move to the high potential region.

**Figure 4:**
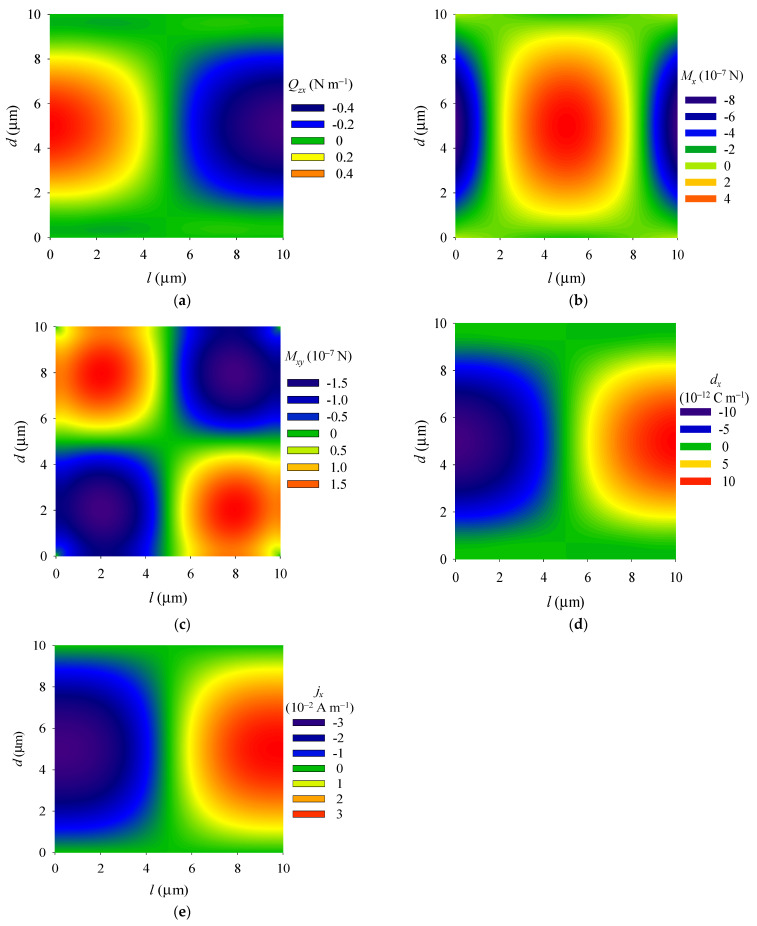
Distributions of (**a**) shear stress *Q_zx_*, (**b**) bending moment *M_x_* (**c**) torque *M_xy_*, (**d**) surface electric charge *d_x_*, and (**e**) surface electric current density *j_x_* in the plate.

The shear stress *Q_zx_*, surface electric charge *d_x_*, and surface electric current density *j_x_* are antisymmetric distribution around the central line *x* = *l*/2 (see Figure 4a,d,e), and extreme values of *Q_zx_*, *d_x_*, and *j_x_* all occur at the central points in the left and right fixed boundaries. The bending moment *M_x_* is symmetrically distributed around the *y*-axis (see Figure 4b). The extreme values also occur at the central points of the left and right fixed boundaries, and the torque *M_xy_* is symmetrically distributed around the diagonal lines of the plate (see Figure 4c).

### 4.2. Effects of Initial Electron Concentration

The effects of the initial electron concentration *n*_0_ on vibrations were examined. With decreasing *n*_0_, numerical solutions of the electromechanical fields converged more easily. When *n*_0_ = 10^14^ m^−3^, the total element number *N_E_* = 150 × 150 is used, as shown in Table 2.

**Figure 5:**
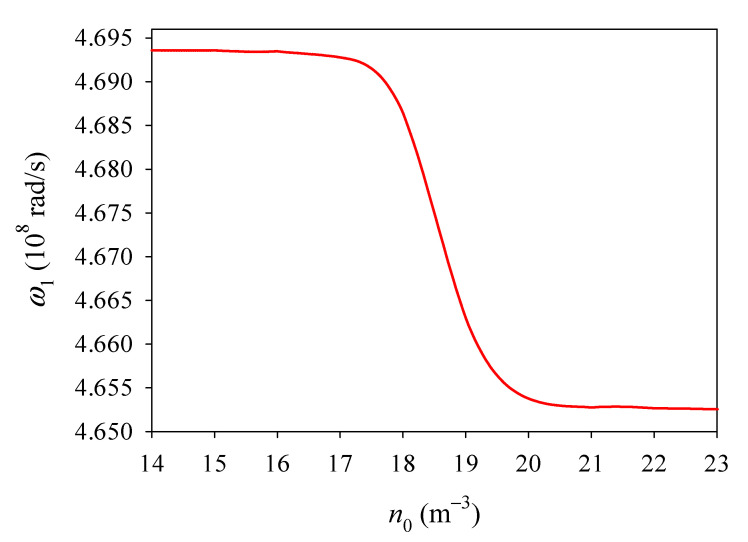
The first natural frequency *ω*_1_ versus initial electron concentration *n*_0_.

The variation in the first natural frequency *ω*_1_ of the PSC plate versus *n*_0_ is plotted in Figure 5. The effect of *n*_0_ on *ω*_1_ is small until it is in the range 10^16^–10^20^ m^−3^, where *ω*_1_ decreases sharply with *n*_0_. Figure 5 indicates an electron screening effect, in which increasing numbers of mobile electrons in the semiconductor will screen the effective polarization charges when *n*_0_ increases. Moreover, Figure 5 indicates that a higher initial electron concentration can reduce the stiffness of GaN PSC.

The distributions of the electromechanical fields along the line *y* = *d*/2 were analyzed. We defined the normalized electron concentration perturbation and the surface electric current density as

(21) $$\Delta \bar{N}=\Delta N/n_{0},\;\;\;\;{\bar{j}}_{x}=j_{x}/\left(qn_{0}d_{11}\right).$$

**Figure 6:**
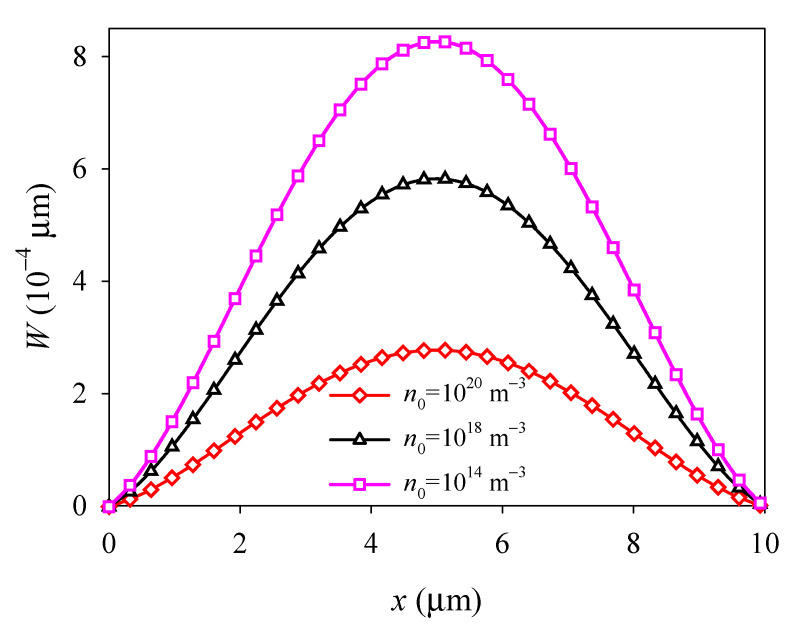
Deflection *W* along the midline *y = d*/2 versus initial electron concentration *n*_0_.

**Figure 7:**
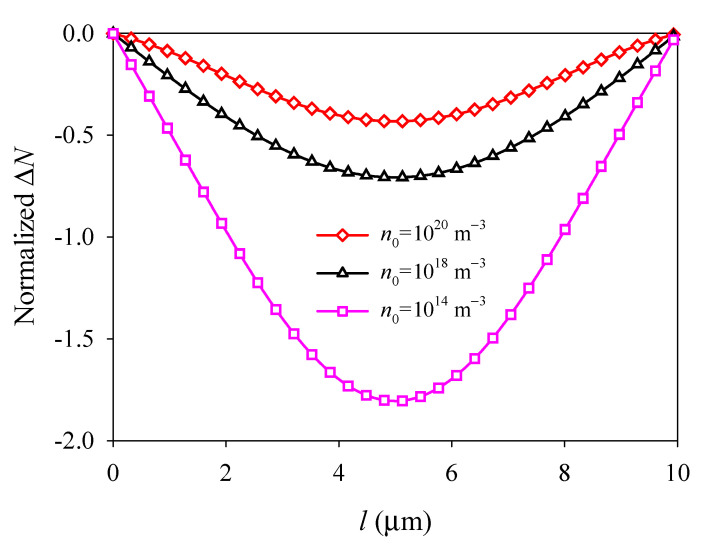
Electric potential *Φ* along the midline *y* = *d*/2 versus initial electron concentration *n*_0_.

**Figure 8:**
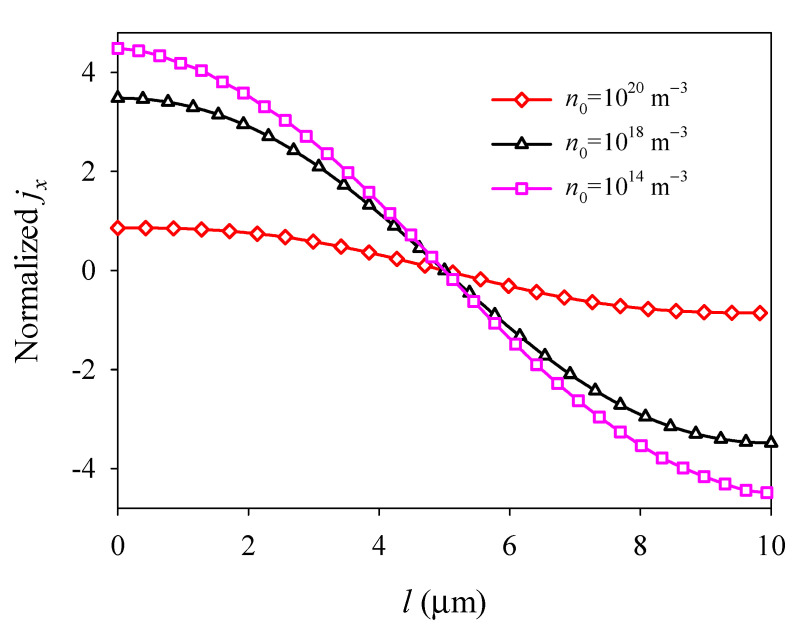
Normalized electron concentration perturbation Δ*N* along the midline *y* = *d*/2 versus initial electron concentration *n*_0_.

With fixed boundaries, the deflection *W*, electric potential Φ, and electron concentration perturbation Δ*N* all exhibit parabolic and symmetric distributions about the central line *x* = *l*/2, and extreme values occur at the central point (see Figure 6, Figure 7 and Figure 8). It is shown that when *n*_0_ increases, *W* and the absolute value of Φ and Δ*N* all decrease with *n*_0_ (see Figure 6, Figure 7 and Figure 8), and when *n*_0_ is relatively large, such as *n*_0_ = 10^18^ m^−3^, Φ and Δ*N* become decrease slowly with *n*_0_ (see Figure 7 and Figure 8). These changes of deflection *W*, electric potential Φ, and electron concentration perturbation Δ*N* with initial concentration n0 all due to the electron screening effect. As *n*_0_ increases, there are more electrons to screen the polarization charge, which leads to a weaker piezoelectric effect and a decrease of the absolute value of Φ and Δ*N*.

**Figure 9:**
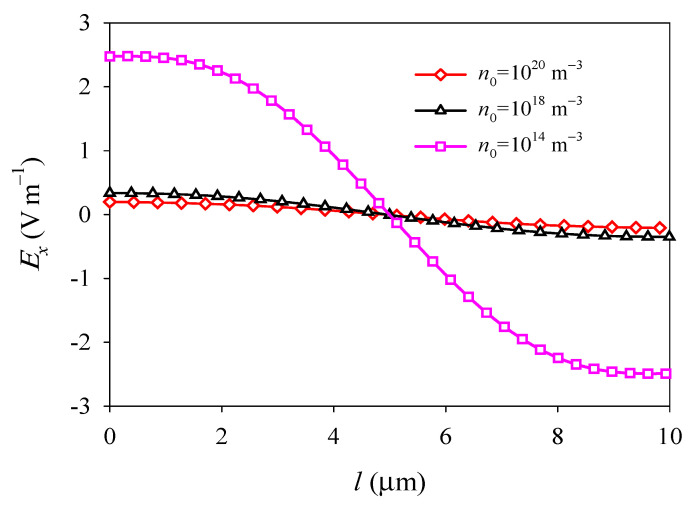
Electric field *E_x_* along the midline *y* = *d*/2 versus the initial electron concentration *n*_0_.

**Figure 10:**
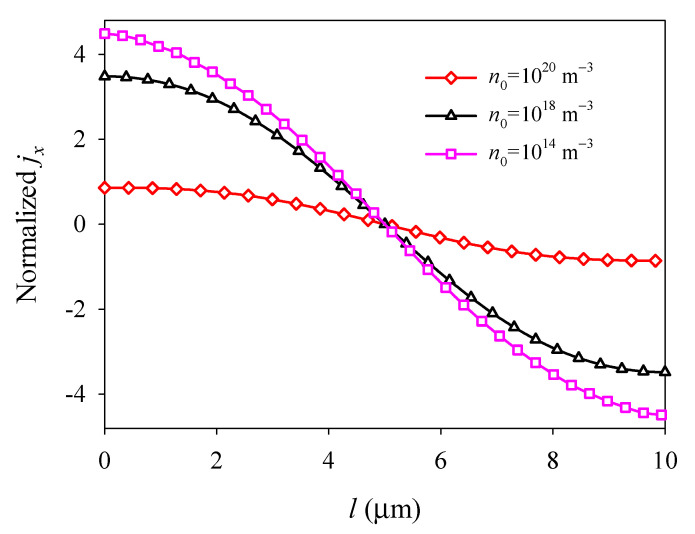
Normalized surface electric current density *j_x_* along the midline *y* = *d*/2 versus initial electron concentration *n*_0_.

The electron field *E_x_* and surface electric current density *j_x_* exhibit nonlinear and antisymmetric distributions about the central line at *x* = *l*/2 and decrease with increasing *n*_0_ because of electron screening (see Figure 9 and Figure 10). It is shown that the initial electron concentration has a significant effect on the electromechanical fields and electronic transport of PSC plates. This phenomenon provides a significant guide for the development and optimization of semiconductors devices, such as nanogenerators, sensors, and field-effect transistors.

## 5. Conclusions

Based on the 3D theory of PSC, the first-order shear deformation theory is extended to develop a simplified 2D plate model for the transient analysis. The flexural vibrations of the structure under a time-harmonic load were investigated with numerical software COMSOL. The vibration modes and natural frequencies of the PSC plate were obtained, and the influence of initial electron concentration on electromechanical behaviors was deeply discussed. The main results from numerical studies can be summarized as follows.

1. The amplitude of the deflection corresponding to the first resonant frequency is much larger than those at higher resonant frequencies, and a high energy conversion efficiency for conversing mechanical energy into electrical energy can be realized in a resonance state;
2. With the increase in the initial electron concentration, the first-order nature frequency decreases until it reaches a constant value. This phenomenon indicates that initial electron concentration plays a role in the stiffness reduction;
3. Due to the electron screen effect, the deflection, electric field, and electric current density in the PSC plate all decrease with the increase in the initial electron concentration.

The size effect has a significant effect on the mechanical and electrical properties of PSC structures at the nanoscale. It is expected that the size effect, such as the surface effect, can be considered in the vibration analysis of PSC plates.

## Figures and Tables

**Table 1:** The elastic, piezoelectric, and dielectric constants of GaN.

Property	Parameter	Value	Unit
Elastic constant	*c*_11_	293.7	GPa
	*c*_12_	124.1	GPa
	*c*_13_	158.5	GPa
	*c*_33_	282.0	GPa
	*c*_44_	22.3	GPa
Piezoelectric constant	*e*_13_	−0.52	C m^−2^
Dielectric constant	*ε*_33_	9.385 × 10^−11^	C V^−1^ m^−1^
Electron mobility	*μ*_11_	9.62 × 10^−2^	m^2^ V^−1^ s^−1^
Diffusion constant	*d*_11_	2.49 × 10^−3^	m^2^ s^−1^

**Table 2:** The values of *W*, *Φ*, and Δ*N* at central point versus the element number *N_E_*.

*N_E_*	*n*_0_ = 10^20^ (m^−3^)			*n*_0_ = 10^14^ (m^−3^)		
	*W* (10^−4^ m)	*Φ* (10^−2^ V)	Δ*N* (10^18^ m^−3^)	*W* (10^−4^ m)	*Φ* (10^−1^ V)	Δ*N* (10^14^ m^−3^)
36 × 36	2.549	6.643	4.020	8.252	8.632	1.796
78 × 78	2.784	6.555	4.307	8.273	8.653	1.802
100 × 100	2.791	6.552	4.316	8.273	8.654	1.802
150 × 150	2.795	6.550	4.321	8.274	8.654	1.802
200 × 200	2.796	6.551	4.321	8.274	8.654	1.802
300 × 300	2.795	6.550	4.321	8.274	8.654	1.802
400 × 400	2.795	6.550	4.321	8.274	8.654	1.802
